# The non-communicable disease e-Cohort for hypertension in Latin America: concept, survey development and study protocol

**DOI:** 10.1080/16549716.2025.2541987

**Published:** 2025-08-26

**Authors:** Patricia J. Garcia, Laura Espinoza-Pajuelo, Hannah H. Leslie, Margaret E. Kruk, Catherine Arsenault, Agustina Mazzoni, Jesus Medina-Ranilla, Javier Roberti, Marina Guglielmino, Ezequiel Garcia-Elorrio

**Affiliations:** a School of Public Health and Administration, Unit of Epidemiology, STI and HIV, Cayetano Heredia University, Lima, Peru; b Division of General Medicine and Geriatrics, Department of Medicine, Washington University in St. Louis, St. Louis, MO, USA; c Division of Prevention Science, Department of Medicine, University of California, San Francisco, CA, USA; d Department of Global Health, Milken Institute School of Public Health, George Washington University, Washington, DC, USA; e Department of Quality, Patient Safety, and Clinical Management, Institute for Clinical Effectiveness and Health Policy, Buenos Aires, Argentina; f Centro de Investigaciones en Epidemiología y Salud Pública, Consejo Nacional de Investigaciones Científicas y Técnicas – CONICET, Buenos Aires, Argentina

**Keywords:** Hypertension, health system performance, chronic disease management, longitudinal data, high-quality health systems

## Abstract

Non-communicable diseases like hypertension cause substantial morbidity and mortality in low- and middle-income countries, where limited access to high-quality care contributes to millions of preventable deaths annually. Traditional assessments of health system performance often rely on structural indicators and cross-sectional, overlooking patient experiences and care processes. In Latin America, amid rising cardiovascular disease, longitudinal tools are needed to guide improvements in healthcare delivery models, particularly for chronic diseases such as hypertension. The NCD e-Cohort is a prospective, longitudinal, mixed-mode (in-person and phone) survey designed to assess health system quality in Latin America. Developed by the QuEST Network, it integrates validated and locally tailored items aligned with the Lancet Global Health Commission’s framework. Key domains include competent care, user experience, health outcomes, confidence in the health, economic impacts, and care pathways. About 600 hypertensive patients aged 35+ will be recruited from sentinel sites in Uruguay (CESCAS) and Peru (CRONICAS), with 300 participants per site. Data will be collected for one year, capturing real-time information on patient journeys and health system performance. This study advances hypertension care assessment by embracing the care-cascade approach and capturing dynamic patient-provider interactions, continuity, and treatment evolution. Although participant attrition may pose challenges, the frequent data collection minimises recall bias. The NCD e-Cohort will generate timely, actionable evidence to inform patient-centred policies and strengthen health systems in the region. Leveraging mobile technology enhances feasibility, scalability, and adaptability, potentially extending this approach to other chronic conditions.

## Paper Context

- **Main findings**: The NCD e-Cohort offers a novel, longitudinal tool that provides detailed, longitudinal insights into the quality of hypertension care, patient experiences, and care pathways in Latin America.
- **Added knowledge**: This study expands understanding of how continuous, real-time, patient-centred data are valuable and can inform improvements in chronic disease management.
- **Global health impact for policy and action**: By identifying systems gaps and guiding evidence-based interventions, these findings can support policymakers and stakeholders in developing strategies to strengthen health systems and improve outcomes in the region.

## Background

Although significant progress has been made in health outcomes, approximately 8.6 million people die each year in low- and middle-income countries (LMICs) due to inadequate access to high-quality healthcare [1]. Nearly 60% of these deaths occur among people who actively sought medical care but did not receive adequate services. Many of these deaths are due to non-communicable diseases (NCDs), including chronic conditions such as hypertension, diabetes, and cancers [2]. Chronic diseases differ from acute conditions in that they require continuous management and multiple healthcare visits, highlighting the importance of chronic care models that adapt to patients’ evolving needs. As these conditions progress over time, health systems must evolve to manage the increasing workload and capacity demands of both patients and providers.

The Lancet Global Health Commission on High-Quality Health Systems introduced the HQSS framework, which integrates structural, process, and outcome measures; moving beyond traditional indicators of equipment and infrastructure to emphasise evidence-based care processes, such as adherence to clinical guidelines for chronic disease management [3–5]. This approach is particularly relevant for chronic disease management in low- and middle-income countries, where coordination and continuity of care for conditions like hypertension remain significant challenges [6]. By balancing structure, process, and outcomes, the HQSS framework offers a more holistic assessment of health system performance and helps identify actionable gaps in chronic care delivery [7].

In Latin America, ensuring coordination and continuity of care for chronic diseases remains particularly challenging. Despite advancements, many health systems are still structured around acute care rather than for sustained management of long-term conditions [8]. Existing evaluations in the region often rely on cross-sectional data. However, hypertension management is inherently dynamic and cyclical: patients may start and stop treatment and alternate between periods of controlled and uncontrolled blood pressure. Recent studies show that longitudinal data are critical for understanding these transitions, monitoring care quality over time, and guiding interventions [9]. Such ongoing measurements support more responsive, patient-centred care and inform evidence-based policies and improve overall health system performance.

In Pampas de San Juan de Miraflores, a densely populated peri-urban district south of Lima, Peru, households are predominantly lower-middle-income, and many residents are migrants from the Andean region. Prior work in this community has documented hypertension and stroke rates well above national averages, underscoring the gap in our understanding of how local health services detect, treat and follow patients through their care pathways [10,11].

Barros Blancos, Uruguay, an urban area with approximately 58,000 residents, similarly faces high burdens of hypertension, obesity, and diabetes. Yet despite these documented prevalence rates, little is known about the processes by which patients navigate the health system, from initial diagnosis through ongoing management [12]. Both sites therefore provide ideal settings to generate the longitudinal care-process data needed to strengthen chronic disease control in Latin America.

To address these gaps, we developed the NCD e-Cohort for hypertension – a longitudinal survey mobile-enabled underpinned by the HQSS framework, designed to evaluate whether high-quality, continuous hypertension care leads to better outcomes. Our primary hypothesis is that patients who receive quality-adjusted care aligned with HQSS principles will achieve higher rates of blood pressure control and improved quality of life compared to those who do not. The e-Cohort leverages rising mobile-phone ownership across the region, offering a flexible tool to track care quality in real time throughout the patient journey.

This longitudinal approach enables ongoing monitoring of care and generates evidence on effective coverage – that is, not just access to services but delivery of high-quality care that meets patient needs over time. By capturing continuous process and outcome data, the e-Cohort will pinpoint key areas for system improvement and inform interventions aimed at closing gaps in chronic disease management [13].

In this paper, we describe our study protocol including survey development, content domains, operational definitions, and implementation strategy. Additionally, we outline our analytical plan for translating findings into actionable policy and practice, with pooled and site-specific analyses designed to generate locally relevant insights.

## Methods

### Aim and study design

The NCD e-Cohort is a longitudinal mixed-mode (in-person and phone) survey that collects near-real-time data on health system quality in selected sites across Latin America. The NCD e-Cohort has four goals: (a) measure both care quality and health system competence in NCD management; (b) describe patient health outcomes, user experience, and care pathways; (c) identify gaps in effective care impacting outcomes; and (d) create a flexible mobile tool for assessing health system performance.

### Survey development process

The survey was developed by the Quality Evidence for Health System Transformation (QuEST) in Latin America and the Caribbean (Peru, Argentina and USA) Network (https://www.redquest-lac.org/) in collaboration with the global QuEST Network, ensuring a regionally relevant tool grounded in the recommendations of the Lancet HQSS Commission. The development group included health system researchers, clinicians, and survey design experts.

We first conducted a scoping review of existing chronic care patient surveys and mapped their quality dimensions into the HQSS framework to identify measurement gaps, particularly in care processes (competent care and systems), user experience, patient-reported outcomes, confidence in the health system and economic impacts [5].

Building on these domains, we drafted the NCD e-Cohort through regular working meetings to ensure clarity, relevance, and alignment with study objectives. We then solicited external peer review from health system experts and survey methods specialists. Additionally, we conducted up to 12 cognitive interviews per site (using community fieldworkers in Peru and Uruguay) to probe comprehension, regional expressions, and cultural appropriateness. Feedback from these steps guided refinements to question wording, response options, and examples, resulting in a culturally tailored, content‐valid tool.

The survey instrument combines existing and previously validated survey questions from past studies and newly developed items. Questions were adapted from the People’s Voice Survey [14], Demographic and Health Surveys (DHS) [15], the Pan American Health Organization (PAHO) HEARTS questionnaire [16], the International Consortium for Health Outcomes Measurement (ICHOM) [17], the Hill-Bone compliance questionnaire [18], the Commonwealth Fund International Health Policy survey [19], the WHO STEPwise approach to NCD risk factor surveillance (STEPS) survey [20], the EQ-5D-3 L survey [21], and the QuEST Network’s Maternal and Newborn Health e-Cohort [22].

Surveys are administered in Spanish, with regional vocabulary adaptations as needed. Data is collected through a mobile phone – based platform using KoboToolbox (https://kobotoolbox.org/), which is configured in Spanish and included built-in logic checks, such as range limits and automated skip patterns. Field supervisors will review data quality weekly and met biweekly with enumerators to address issues.

The instrument covers the major HQSS domains – competent systems, competent care, user experience, health outcomes, confidence in the health system, and economic outcomes – and an additional domain of care pathways [5]. The next sections describe each domain’s measures.

#### Competent systems: continuity and integration

Competent systems reflect how effectively healthcare services support sustained, coordinated, and integrated care delivery over time. In the context of hypertension management, these systems ensure consistent and complementary healthcare delivery, enhancing patient outcomes through relational continuity (regularly seeing the same provider), informational continuity (providers having consistent access to medical history, test results, and ongoing treatment), and integrated care across multiple levels and settings.

These aspects are measured monthly from the patient’s perspective via structured phone surveys. Participants provided detailed reports on the number and type of healthcare providers they have consulted, frequency of visits with the same provider, and whether new providers were aware of their medical history and treatment plans. Additionally, surveys capture the types of healthcare facilities visited, reasons for visits, and experiences of care integration or fragmentation [23]. Patients who did not seek care also indicate their reasons, such as financial barriers, transportation issues, or dissatisfaction with previous care.

Analysing this data will reveal whether relational continuity is associated with improved hypertension control. Identifying disruptions in continuity and integration will also enable targeted interventions to enhance seamless care delivery, thereby strengthening overall system competence.

#### Competent care

Competent care focuses on the quality of clinical decision-making and service provision, including whether providers assessed cardiovascular risk, ordered appropriate tests, prescribed appropriate medications, and scheduled appropriate follow-up care.

We incorporate questions from the HEARTS 10-year cardiovascular risk assessment to estimate risk of myocardial infarction, stroke, or cardiovascular death based on individuals profiles [13]. HEARTS tailored this tool to Latin American settings, integrating relevant regional and national hypertension guidelines to enhance clinical relevance [24].

At baseline and monthly follow-ups, participants report on clinical care received: whether healthcare providers assessed their clinical history, conducted recommended exams or tests, provided guideline-adherent treatments, and arranged follow-up. These self-reported measures span the continuum of care (see Table 1 for examples). All competent care items are assessed from the patient’s perspective with no record verification.

**Table 1.** Example of competent care items measured in the NCD e-Cohort across the continuum of care.

**Evaluation of pre-existing conditions**	
● Previous diagnosis of hypertension ● Previous diagnosis of diabetes ● Previous diagnosis of hypercholesterolemia	● Previous chronic kidney disease ● History of heart attack or chest pain from heart disease or stroke
**CVD risk estimation**	
● Current CVD (coronary artery disease, cerebrovascular disease, or peripheral vascular disease) ● Current chronic kidney disease ● Current diabetes	● Hypercholesterolemia ● BMI ● Active tobacco use ● Sex and age ● Systolic blood pressure
**Patient Factors and Health Behaviors**	**Clinical Examinations and Diagnostic Tests**
● Patient activation levels ● Beliefs about medications ● Mobility level ● Level of self-care (ability to bathe or dress self) ● Ability to perform usual activities normally ● Presence of pain/discomfort ● Level of anxiety/depression ● Salt consumption levels ● Frequency of junk food consumption ● Presence of physical activity ● Alcohol consumption	● Blood test ● Urine test ● Electrocardiogram ● Echocardiogram
**Regular check-ups and monitoring**	
● Healthy lifestyle modifications ● Salt consumption reduction ● Alcohol consumption reduction ● Tobacco use reduction ● Pharmacological treatment/modification ● Presence of telephonic or virtual monitoring	● Number of consultations in the last month ● Continuity of care with regular physician ● Continuity of care with regular place of care ● Next appointment schedule
**Patient education and emergency preparedness**	
● Effective communication of the diagnosis and awareness of the impact on the patient’s welfare	● Report of adverse effects of medication ● Awareness of adherence to treatment

In the analysis, we will examine whether receiving quality-adjusted care (a composite of competent care elements) is associated with hypertension control after one year. We will also explore if certain care patterns (like continuity with the same provider) lead to better health outcomes.

#### User experience

Understanding users’ experience is vital for assessing care quality and identifying areas for improvements in patient-centred services. The survey engages individuals with hypertension to capture their experiences throughout their care journey, covering aspects such as the level of respect and empathy received from healthcare providers, the clarity of information and guidance provided, wait times and overall interaction length, and any experiences of discrimination. Questions are adapted from validated patient-experience surveys. We also ask about the support received during visits and adequacy of pain relief, capturing comfort and care quality.

We will compare one-year blood pressure control between patients with positive vs. negative care experiences (stratified by baseline cardiovascular risk level). We also assess whether longer consultation times correlate with higher patient satisfaction.

#### Health outcomes

We conduct a comprehensive follow-up to monitor health outcomes. Blood pressure control status is recorded at baseline and endline to track hypertension control over the one-year study period. The survey also incorporates patient-reported outcome measures (PROMs), covering self-rated health and health-related quality of life (HRQoL) assessed through the EuroQol five-dimension scale questionnaire (EQ-5D-3 L), as well as psychological well-being and other challenges of managing chronic conditions, ensuring a holistic approach.

In the analysis, we examine changes in EQ-5D-3 L health dimensions over the year between patients receiving quality-adjusted care and those who do not, stratified by risk classification. We will explore whether improvements in HRQoL correlate with higher care quality.

#### Confidence in the systems

Patient confidence in the healthcare system influences health-seeking behaviours and treatment adherence. Confidence is measured at baseline and endline to minimise respondent burden, but monthly data on care-seeking and facility choice serve as indirect indicators of trust in the system. Questions assess patient trust in providers and facilities, and whether this trust change over time. During follow-up calls, we ask if participants would recommend their current facility to others as a proxy for satisfaction and/or trust.

Analysis of these perceptions over time will test if confidence relates to health outcomes. We will examine whether those with higher satisfaction and willingness to recommend their facility achieve better hypertension control and will use these insights to suggest health systems improvements.

#### Economic benefits

Access to affordable care is crucial, especially for chronic conditions like hypertension [25–27]. Recognising that high-quality healthcare should mitigate the risk of financial hardship, the survey records all health expenditures incurred by participants, covering medical consultations, diagnostic tests, medications, transportation, and additional related expenses such as travel or special dietary needs.

Participants provide information about financial sources used to cover their healthcare expenses. They report whether costs were covered by income, insurance, donations, or borrowing money. The survey also identifies patients who avoided seeking care due to cost concerns and explores underlying reasons. We will analyse economic barriers in relation to health outcomes to pinpoint policy interventions. Understanding the financial burden of care helps inform strategies for making hypertension care affordable and accessible.

#### Care pathways

Understanding where patients seek care (the sequence and choice of healthcare contacts) is vital for optimising healthcare delivery. Participants report the healthcare facilities visited and reasons for their choices. The survey assesses whether their usual source of care is covered by health insurance or if there is a mismatch, and if individuals supplement public healthcare with private services.

We can identify common care pathways and factors influencing facility choice. We will explore, for instance, how insurance mismatches may affect outcomes. Insights from this analysis will highlight opportunities to improve how patients navigate the health system, ultimately enhancing care continuity and patient satisfaction.

Table 2 maps our novel research and policy-relevant questions related to the above HQSS framework domains.

**Table 2.** Novel research and policy-relevant questions mapped to HQSS domains.

Quality Domain	Research Question	Key Variables Included in Analysis	Assessment Timing	Data Collection Mode
Competent care and systems	What is the difference in the proportion of patients achieving hypertension control after one year between those who receive quality-adjusted care and those who do not, stratified by risk classification?	Health outcome (control) and processes care (quality-adjusted care)	Monthly follow-up and endline home visit	In-person and phone
	Is there an association between baseline blood pressure and the probability of seeking health care? If so, is baseline blood pressure a confounder of the association between health care and control of hypertension?	Baseline blood pressure, number of visits, and health outcomes	Baseline home visit and monthly follow-up	In-person and phone
	Do patients who initiate or modify their treatment at their first visit achieve better health outcomes than those who do not?	Medication and health outcomes	Baseline home visit and monthly follow-up	In-person and phone
	Do patients with more than one visit with the same physician achieve better health outcomes than those who see different physicians?	Continuity and health outcomes	Monthly follow-up	Phone
Userexperience	What is the difference in the proportion of patients achieving hypertension control after one year between those with a positive user experience and those without, stratified by risk classification?	User experience and health outcomes	Monthly follow-up	Phone
	Are patients with longer consultation times or more satisfied with their care achieved better health outcomes?	Consultation length and satisfaction, health outcomes	Monthly follow-up	Phone
Health outcomes	How does the change in the quality of life over one year differ between patients who receive quality-adjusted care and those who do not, stratified by risk classification?	EQ5DL health dimensions and health outcomes	Baseline and endline visit	In-person
Confidence in the system	What are the trends in patient confidence in the healthcare system – measured by satisfaction and likelihood to recommend – over the course of the follow-up?	Satisfaction and recommendation level	Monthly follow-up	Phone
Economic benefits	What proportion of patients did not seek care due to cost concerns?	Reasons for not seeking healthcare	Monthly follow-up	Phone
Care pathways	Where do patients with hypertension typically seek care, and what factors influence their choice of healthcare facility?	Usual place of care	Monthly follow-up	Phone
	How does mismatch between a patient’s health insurance and their usual place of care influence their health outcomes?	System coverage and health outcomes	Baseline and endline visit	In-person

### Sample size and respondent selection

Two sentinel sites will be selected in two countries: an urban setting in Barros Blancos, Uruguay, and a peri-urban setting in Pampas de San Juan de Miraflores, Lima, Peru. Both sites have hosted prior cohort studies on non-communicable diseases (the CESCAS cohort study in Uruguay, focused on cardiovascular disease and its risk factors in the Southern Cone; and the CRONICAS cohort study in Peru, focused on chronic non-communicable conditions), making them ideal for examining healthcare processes for hypertension and related cardiovascular conditions. The target population includes adults aged 35 and older who report having been diagnosed with hypertension by a health professional and/or have an elevated blood pressure reading (≥140/90 mmHg) at the time of enrolment. Recruitment will be conducted in person, with home visits in Peru and through a research centre in Uruguay, ensuring accessibility and representation across both sites.

Eligible participants must be adult residents of the community (35 years or older), with access to a mobile phone, and without plans to relocate within the next 12 months.

We aim to enrol 600 adults with uncontrolled hypertension (systolic blood pressure ≥140 mmHg or diastolic blood pressure ≥90 mmHg) — 300 at each sentinel site (CRONICAS-Peru and CESCAS-Uruguay). Evidence from different settings shows that approx. 30%–45% of patients receive guideline-adherent, high-quality care [28,29]. Conservatively, we therefore assume that 30% of participants at each site (≈90/300) will receive high-quality, quality-adjusted care, with the remaining 210 managed with lower-quality care. Drawing on published gaps in control by care quality, we power each within-site comparison to detect a 15-percentage-point (pp) absolute difference in 12-month blood-pressure control (e.g. 25% vs 10%) – a magnitude smaller than reported in a previous uncontrolled-hypertension cohort (22.6 pp) [29]. With unequal allocation (*n* = 90 vs 210) and α = 0.05 (two-sided), each site’s sample of 300 provides ≈ 85% power to detect a 15pp difference in blood pressure control. Allowing for 15% attrition would maintain power ≈ 79%, meeting our ≥80% criterion before attrition and preserving precision for secondary subgroup analyses.

#### Screening and recruitment log

We will characterise individuals who declined participation or were unreachable through a detailed screening and recruitment log, which captures basic demographics (sex, age band), screening blood pressure, and neighbourhood deprivation. Logistic regression models predicting participation will help quantify potential selection bias, and inverse-probability-of-participation weights will be incorporated into secondary analyses when substantial bias is detected.

### Data collection

**Figure 1.**
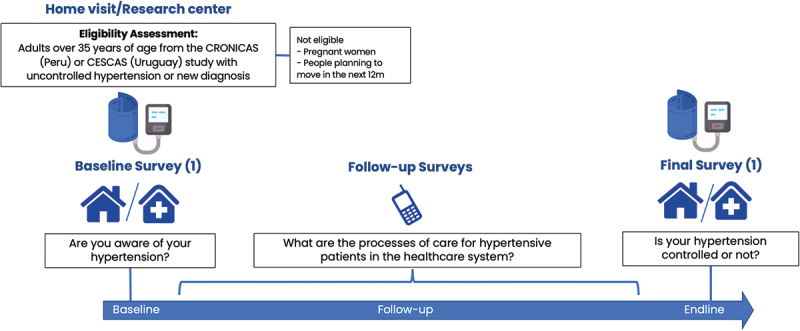
Structure of non-communicable disease E-Cohort study for hypertension.

The data collection for the NCD e-Cohort Study begins with an eligibility assessment conducted at a participant’s home or a research centre. This assessment targets adults over 35 years who report a prior hypertension diagnosis by a doctor and/or have uncontrolled hypertension confirmed at the visit. Exclusions include pregnant women and individuals planning to relocate within the following year. Once eligible participants are identified, they complete a baseline survey focused on their awareness of their health/hypertension status and usual place of care. This survey establishes foundational data on participants’ knowledge of their health condition (Figure 1).

Following the baseline, participants undergo a series of monthly follow-up surveys conducted by phone. These surveys capture where participants seek care for their NCDs, the reasons for their healthcare choices, and indicators of competent care and user experience (described above). At the study’s end, a final survey is conducted to assess the control status of participants’ blood pressure providing a comprehensive picture of health outcomes and care effectiveness over the study period.

Each monthly phone interview is designed to take approximately 20 minutes, while the baseline and endline in-person surveys require up to 40 minutes. To reduce respondent fatigue, questions are grouped into concise modules and interviewers use a conversational style to maintain engagement.

We mapped each HQSS domain to specific data collection timepoints. Competent care, competent systems, user experience, and economic burden are assessed monthly through follow-up phone surveys. Health outcomes and confidence in the health system are measured at baseline and endline, with blood pressure control evaluated in-person. Certain dimensions – particularly competent care and user experience – are measured repeatedly in response to patients’ self-reported healthcare interactions. This design results in a panel dataset with repeated observations per participant over the 12-month follow-up period.

To support retention across the entire 12-month mobile follow-up, participant receive a free airtime top-up (mobile credit) or a one-time 11 USD supermarket voucher upon finishing all scheduled monthly surveys.

While the mixed-mode (in-person and phone) design enhances feasibility, it may introduce biases due to variable mobile phone ownership, inconsistent network coverage, and potential underrepresentation of individuals in remote or underserved areas. To mitigate this, the study includes initial in-person recruitment and home visits to maximise inclusion and data quality.

To minimise recall bias, the survey collects information at frequent monthly intervals, reducing reliance on long-term memory. Standardised prompts and short recall periods are used during phone interviews. To enhance retention, multiple contact attempts are made at different times of day, and participants receive reminder calls and clear instructions about the importance of their continued participation.

### Comparison group definitions

Our primary analysis will evaluate whether patients receiving quality-adjusted care – defined as HEARTS guideline-concordant care – achieve better hypertension control (systolic blood pressure < 140 mmHg and diastolic blood pressure < 90 mmHg) at 12 months compared to those who do not.

Quality-adjusted care is operationalised using five key indicators: (1) ≥3 hypertension visits per year; (2) blood pressure measured at every visit; (3) comprehensive lifestyle and treatment discussions; (4) annual cardiovascular disease risk screening; and (5) therapeutic adjustments following guidelines. Patients meeting all five criteria are classified as receiving quality-adjust care.

### Analysis plan

#### Baseline balance and adjustment

To ensure robustness in estimating the effects of care quality, we will rigorously assess baseline comparability across exposure groups (people receiving quality-adjust care vs. the ones who do not, high vs. low continuity, positive vs. negative user experience). A comprehensive first table will summarise key demographic (age, sex), socioeconomic (education, household wealth), and clinical (baseline blood pressure, comorbidity count) characteristics as well as health insurance status. Standardised mean differences exceeding 0.10 will trigger covariate balancing using inverse-probability-of-treatment weighting (IPTW). Primary effect estimates will be reported as unadjusted, regression-adjusted, and doubly robust (IPTW combined with regression adjustment).

#### Main analysis

Logistic regression models, weighted and adjusted as described, will estimate associations between quality-adjusted care, continuity of care, user experience, and hypertension control. Marginal risk ratios and differences, including their 95% confidence intervals, will be reported to provide intuitive and actionable estimates for policymakers.

Longitudinal patient-reported outcomes, particularly changes in EQ-5D-3 L dimensions of health-related quality of life, will be analysed using mixed-effects linear models stratified by cardiovascular risk. Patient confidence in health systems will be measured at baseline and endline, with repeated-measures analyses evaluating trends in patient satisfaction and likelihood-to-recommend ratings throughout the year.

#### Sensitivity analysis

We will conduct several robustness checks to strengthen our primary analyses. These checks will include: (1) employing alternative thresholds to define quality-adjusted care and user experience; (2) trimming extreme weights to assess the stability of our findings; and (3) applying entropy balancing if extreme weights are identified. Such comprehensive sensitivity analyses will ensure our results are robust to multiple analytic choices.

Additionally, we will evaluate whether continuity of care – categorised as high if participants have three or more visits with the same provider within 12 months – and positive user experience – assessed through eight patient-reported indicators including satisfaction, communication, timeliness, respect, and trust – independently predict blood pressure control at 12 months.

Although blood pressure measurements are limited to baseline and endline due to logistical constraints, detailed monthly follow-up data on intermediate care processes such as medication initiation, adherence, and counselling will be available. We will leverage these monthly phone follow-ups to perform sensitivity analyses examining self-reported outcomes at 6 months, providing an additional timepoint comparison to the primary 12-month outcomes.

Finally, to assess robustness against missing data, we will conduct sensitivity analyses including complete-case analyses, delta-adjusted pattern-mixture models, and inverse-probability-of-censoring weights, thereby evaluating the impact of various assumptions regarding data missingness.

#### Secondary analysis

Secondary analyses will compare findings across sites to explore potential differences in care experiences and outcomes. We will descriptively benchmark key indicators against available national-level data thus contextualising our results and aiding interpretation regarding generalisability to similar Latin American settings.

Economic outcomes, such as the frequency of care-avoidance due to cost and detailed expenditures reported monthly, will be described to understand barriers to high-quality care. Analysis of patient-reported care pathways will investigate factors influencing healthcare choices, mismatches between insurance coverage and preferred providers, and the influence of economic constraints on healthcare access and continuity.

Ultimately, the data from the NCD e-Cohort for hypertension study can serve multiple purposes – tracking quality of care, benchmarking healthcare performance across regions, guiding clinical training initiatives, informing resource allocation, and standardising quality metrics across health systems.

#### Approach to missing data

Recognising potential challenges due to incomplete data from monthly mobile follow-ups, we will systematically address missingness. Patterns of missing data will be assessed visually and statistically (Little’s MCAR test). Missing data will be imputed through multiple imputations by chained equations under a missing-at-random assumption, utilising 20 imputed datasets that incorporate all relevant outcome, exposure, and auxiliary variables.

## Discussion

The NCD e-Cohort represents a substantial advancement in evaluating and improving the quality of care for individuals with chronic conditions like hypertension in LMICs, particularly in Latin America. Many countries face an epidemiological transition characterised by increasing NCD burdens without health systems fully equipped for continuous, patient-centred care [30,31]. This gap highlights the need for innovative approaches to health system assessment and improvement [5,24].

Unlike cross-sectional surveys such as DHS or WHO STEPS, which provide only point-in-time snapshots, the NCD e-Cohort enables longitudinal monitoring of patients’ experiences, outcomes, and health systems interaction. This design captures trends, breakdowns, and improvement over time – key insights that single-time surveys may miss.

Integrating mobile technology in data collection is another significant contribution of the NCD e-Cohort. Leveraging widespread mobile phone use in the region enhances feasibility and scalability, enabling continuous, real-time data collection. This improves the accuracy and timeliness of information on patient with geographical, financial, or mobility constraints. However, blending in-person and telephone interviews may introduce mode effects (differences in responses by interview mode). Future studies should incorporate calibration exercises to ensure data comparability across modes.

The NCD e-Cohort’s development was collaborative and regionally grounded, ensuring cultural relevance. By combining validated items from established instruments with new, locally tailored questions, the survey comprehensively addresses key variables pertinent to chronic disease management in Latin America. The focus on domains such as competent care, user experience, health outcomes, confidence in the health system, and economic outcomes aligns with the HQSS framework, providing a robust structure for health system assessment [5].

A critical added value of the NCD e-Cohort is its emphasis on patient-reported outcomes and experiences. Patients are not just care recipients but also key informants on care quality. Incorporating their perspectives on provider communication, care coordination, respect, and trust captures dimensions often overlooked by traditional assessments. Understanding these factors is essential for identifying areas where health systems may fall short and developing interventions that enhance patient satisfaction and engagement.

By incorporating data elements from the HEARTS initiative [16], our tool gains a robust clinical perspective on quality improvement. Synthesising insights from HEARTS and similar initiatives provided a multifaceted view of health interventions impacts. Additionally, patient interactions fluctuate due to system fragmentation, changing personal needs, or external disruptions, highlighting the value of longitudinal tracking.

The longitudinal nature of the NCD e-Cohort also allows for the examination of the relationship between care processes and health outcomes over time. This ability to link processes with outcomes longitudinally is invaluable for identifying factors influencing blood pressure control and quality of life. This information helps healthcare providers and policymakers implement evidence-based strategies to improve hypertension management [32].

We emphasise that our findings will be specific to the two sentinel sites rather than nationally representative. Analyses will pool data with site-fixed effects to account for contextual differences and explore outcome variations between sites. Although results are not nationally generalisable, the selected urban and peri-urban sites are relevant, providing transferable insights for similar settings regionally. Future research could benchmark key indicators against national surveys to gauge broader applicability.

To support long-term implementation of the e-Cohort approach, infrastructure such as mobile connectivity, data storage, and trained personnel are required. With this infrastructure, the platform presents opportunities to embed implementation research, providing real-time quality improvement feedback. Additionally, future studies should investigate survey mode effects and consider multi-modal expansions (like web or SMS) to enhance respondent flexibility and data quality.

The NCD e-Cohort generates detailed, longitudinal data that can directly inform policy and system reform. Identifying workforce shortages, medication stock-outs, and financial barriers enables policymakers in resource-limited settings to prioritise investments in training, continuity-of-care interventions, and affordable hypertension medications. Tracking metrics across regions and time fosters accountability. Such benchmarking supports evidence-based, scalable quality improvement initiatives tailored to local constraints.

Despite our efforts, patient self-report data may carry risks of recall bias and interpretation variability across diverse linguistic groups, even with careful cultural adaptation. Additionally, longitudinal phone-based follow-up may encounter inconsistent engagement due to phone ownership or network stability, potentially introducing selection bias and limiting generalisability. Mitigation strategies like frequent follow-ups and tailored reminders aim to enhance retention and reduced recall bias, particularly in mobile-based surveys.

Conversely, the NCD e-Cohort offers a novel, patient-centric perspective across the healthcare journey, emphasising patient experiences and outcomes. Incorporating validated surveys questions and adaptable design enhances data robustness. The innovative methodology aligns with global health evaluation frameworks and presents a scalable model for assessing longitudinal health system performance. The anticipated comprehensive data have significant potential to influence policy and practice, promoting responsive, patient-centred care regionally.

In summary, the NCD e-Cohort offers an innovative, scalable, and policy-relevant approach to monitoring and improving hypertension care in Latin America. Its longitudinal and patient-centred design captures the nuances of real-world care experiences, enabling both rigorous research and practical system improvements.
